# Croupy Season

**Published:** 1867-02

**Authors:** 


**CROUPY SEASON.**—In the early part of spring many children die of croup, which is simply a common cold settling itself in the windpipe and spending all its force there. Why it should tend to the throat in them, rather than to the lungs as in some grown persons, and to the head of others, giving one man influenza, another pleurisy, a third inflammation of the lungs, and a fourth some low form of fever, is not so important as to know the causes of croup and the means of avoiding it. The very sound of a croupy cough is perfectly terrible to any mother who has ever heard it once. In any forty-eight hours, it may carry a child from perfect health to the grave. Croup always originates in a cold, and in nine cases out of ten this cold is the result of exposure to dampness, either of the clothing or of the atmosphere, most generally the latter, and particularly that form of it which prevails in thawy weather, when snow is on the ground, or about sun-down in the early spring season. At mid-day the bright sun lures the children out of doors, and having been pent up all winter, a hilarity and a vigor of exercise are induced, much beyond what they have been accustomed to recently. They do not feel either tired or cold; but evening approaches, the cool of which condenses the moisture contained in the air, this rapidly abstracts the heat from the body of the child, and with a doubly deleterious impression; for not only is the body cooled too quickly, but by reason of the previous exercise, it has been wearied and has lost a great deal of its power to resist cold, hence the child is chilled. Exercise has given it an unusual appetite, a hearty supper is taken, and in the course of the night the reäction of the chill of the evening before sets in, and gives fever; the general system is oppressed, not only by the hearty meal, but by the inability of the stomach to digest it, and fever, oppression, and exhaustion all combined, very easily sap away the life of the child. In fact, it may yet be found, when the nature of diphtheria is better known, that it is a typhoid croup, malignant croup.

Children should be kept as warmly clad, at least until May, as in the depth of winter; they should not be allowed to remain out of doors later than sun-down, when they should be brought into a warm room, their feet examined and made dry and warm, their suppers taken, and then sent to bed, not to go outside the doors until next morning after breakfast. All through February, March, and until the middle of April, especially when snow is on the ground, children under eight years of age should not be allowed to be out of doors at all, later than four o'clock in the afternoon, unless the sun is shining, or unless they are kept in bodily motion so as to keep off a feeling of chilliness. We have never lost a child, but feel that it must be a terrible calamity. Young mothers seldom get over the loss of a first born. Surely, then, it is worth all the care suggested in this article, to avert a calamity which is to be felt until we die. The commonest sense dictates the instant sending for a physician in case of an attack of croup, but the moment a messenger is dispatched, have three or four flannels, dip them in water as hot as your hand can bear, and apply them successively to the throat of the child, so as to keep the throat hot all the time, so as to evaporate the matters, which if retained, cause the clogging up inside which soon stops the breath. Hot water should be constantly added to that in which the flannels are thrown, so as to keep it all the time hot. Keep the water from dribbling on the clothing of the child, and see to it that the feet are dry and warm. Most likely the child will be out of danger before the physician arrives, and it is pleasant to be able to turn over the responsibility on him. Loose cough, freer breathing, and a copious discharge of phlegm indicate relief and safety.

Croup seldom comes on suddenly. Generally it has at first no other symptoms than those of a common cold, but the very moment the child is seen to carry his hand towards the throat, indicating discomfort there, it should be considered an attack of croup, and should be treated accordingly. When a child is sick of any thing, no physician can tell where that sickness will end. So it is with a cold, it may appear to be a very slight one indeed, still it may end fatally in croup, putrid sore throat, or diphtheria. The moment a mother observes croupy symptoms in a child from two to eight years, the specially croupy age, arranges to keep it in her own room, by her own side, day and night, not allowing it for a moment to go outside the door, keeping the child comfortably warm, so that no chilliness nor draft of air shall come over it. Light food should be eaten, no meats or hot bread, or pastries. The whole body, the feet especially, should be kept warm all the time. Rubbing twenty drops of sweet oil into the skin over the breast, patiently with the hand, two or three or more times a day, often gives the most marked relief in a cold, thus preventing croup from supervening on an attack of common cold. Such a course promptly pursued will promptly cure almost any cold a child will take, and will seldom fail to ward off effectually, in a day or two, what would otherwise have been a fatal attack of croup, with its ringing, hissing, barking sound, and its uneasy, oppressive, and labored breathing, none of which can ever be mistaken when once heard. Many a sweet child is lost thus, the parents are aroused at dead of night with a cough that suggests croup; but it seems to pass off, and in the morning they wake up with a feeling of thankful deliverance from a boding ill. The child runs about all day as if perfectly well; but the next night the symptoms are more decided, and on the third night the child dies; but this would have been averted with great certainty, if from the first night, the child had been kept in a warm room, warmly clad, the bowels had been kept free; and nothing had been eaten but toast with tea, or gruel or stewed fruits.

